# Pelagic Larval Duration and Isolation by Distance in Coastal Species

**DOI:** 10.1111/eva.70187

**Published:** 2025-12-09

**Authors:** Atal Saha, Per Erik Jorde, Marte Sodeland, Lars Mørch Korslund, Halvor Knutsen

**Affiliations:** ^1^ Department of Natural Sciences, Centre for Coastal Research (CCR) University of Agder Kristiansand Norway; ^2^ Institute of Marine Research Flødevigen Research Station Bergen Norway

**Keywords:** local adaptation, marine genomics, North Atlantic, snails, SNP

## Abstract

Dispersal among populations is crucial both for demographic stability and for the evolutionary potential of species. In marine organisms, dispersal has been shown to be prevalent during pelagic early life stages. Consequently, pelagic larval duration (PLD) has been proposed as an important driver of gene flow in marine species and is influencing genetic differentiation among populations. Despite this prediction, empirical studies have often failed to find the expected correlation between PLD and genetic metrics of gene flow. This could mean either that PLD is a poor predictor of gene flow or that differences in methodology, oceanography or sampling design across studies obscure the underlying mechanisms of gene flow. In the present study, we address these issues by using a consistent sampling design for 10 coastal species with previously published genetic data (ddRAD and microsatellites), and that differ in PLD. We investigate gene flow using an isolation‐by‐distance (IBD) model with pairwise *F*_ST_‐estimates regressed against distances measured along the prevailing coastal ocean current in the study region. We find a significant (*p* < 0.05) correlation between species' PLD and IBD slopes, with a moderately strong correlation (*r* ^2^ > 0.5), These findings support the notion of PLD as a key factor determining dispersal and gene flow among populations of coastal species. Our findings reiterate genetics as a useful tool for inferring population dispersal in marine organisms when potentially confounding factors are eliminated by adopting a consistent sampling design.

## 1 Introduction

The extent of dispersal and associated gene flow among populations is crucial both for the demographic stability of populations on ecological timescales and the long‐term evolutionary potential of species (Bullock et al. 2006; Faurby and Barber 2012). Estimating dispersal rates between populations, and understanding the modes of dispersal, i.e., whether active or passive, and whether occurring as seeds, eggs, larvae or adults, remains a significant area of interest (Bullock et al. 2002; Green and Sánchez 2006). Despite its importance for ecological and evolutionary processes, as well as for management and conservation (Cowen et al. 2006), comprehensive knowledge of dispersal mechanisms is still lacking for many species. With degrading coastal habitats and declining fishery stocks there is an urgent need to understand how modes of dispersal affect gene flow and spatial patterns of intraspecific diversity of species. (Cowen et al. 2006; Henriksson et al. 2025; Norderhaug et al. 2024).

Degree of reproductive isolation and extent of dispersal and gene flow among populations in the dynamic marine environment is influenced by various interacting processes As most marine organisms have larvae that are small in size and pelagic in nature, oceanographic processes are thought to have a significant role in shaping dispersal and thus genetic structure (Faurby and Barber 2012; Hellberg 2009). In particular, it has long been assumed that longer pelagic larval duration (PLD) results in greater dispersal potential, which eventually should result in higher gene flow and weaker genetic differentiation within species (e.g., Bohonak 1999; Roberts 1997). Nevertheless, significant genetic structuring has been reported in many marine species, even those that have pelagic larval stages and/or are highly mobile as adults (e.g., Hauser and Carvalho 2008; Knutsen et al. 2003), and several studies have reported weak or no correlation between genetic differentiation and PLD (e.g., Galarza et al. 2009; Riginos et al. 2011; Weersing and Toonen 2009). For species with limited dispersal ability, a correlation between genetic and geographic distance may be evident (i.e., isolation by distance, IBD: Slatkin 1993). Overall, the relationship between dispersal potential and realized gene flow remains complex and species‐specific, reflecting the interplay between life‐history traits and oceanographic variability.

In addition to distance, the extent of gene flow can be limited due to specific life history traits (e.g., Whiting et al. 2022), sex‐biased dispersal (e.g., Hutchings and Gerber 2002), bathymetric boundaries (e.g., Catarino et al. 2015), temperature (e.g., Stanley et al. 2018) and salinity (e.g., Johannesson and André 2006; Johannesson et al. 2020), which may explain why significant genetic heterogeneity has been observed in species with apparently high potential for gene flow. Because of these multitudes of factors influencing species' genetic structure, elucidating the role of gene flow in marine organisms in general is a complex task and a multispecies comparative approach can be beneficial (Gagnaire 2020; Waples 1987). The lack of a multispecies perspective is presently a shortcoming of nearly all empirical studies of connectivity (Toonen et al. 2011; Treml et al. 2012) and population genetics/genomics. For instance, in a recent comprehensive review of studies of genetic population structure within the Baltic Sea only one study out of more than 200 dealt with multiple species (cf. Wennerström et al. 2017). A multi‐species study in a common geographic region and utilizing a uniform sampling plan is a promising approach to limit influences of confounding factors when exploring the species' different dispersal potentials (e.g., Nuñez‐Penichet et al. 2022). In particular, a common study region serves to maintain a measure of uniformity among study organisms in potential dispersal routes, including ocean currents.

There have been several studies aimed at testing hypotheses related to the duration of PLD and geographic scales of population connectivity as inferred from genetic data. While earlier studies often gave conflicting results on the relationship between PLD and genetic estimates of dispersal, a review and reanalysis of available data (Selkoe and Toonen 2011) revealed that these inconsistencies often stemmed from inappropriate sampling scales: either too narrow to detect population structure or too broad, spanning multiple biogeographic regions. In the present study, we test the hypothesis that pelagic larval duration (PLD) is an important factor in determining the amount and extent of gene flow in marine organisms. Because gene flow is not directly observable, especially at pelagic early life stage(s), we do this by testing if PLD correlates negatively with observed IBD, i.e., the slope of linearized *F*_ST_/(1−*F*_ST_), against geographic distance. We repeat this across 10 coastal species sampled from the same coastal range, using published data on ballan wrasse ( *Labrus bergylta* : Seljestad et al. 2020), black goby (*Gobus niger*: Catarino et al. 2022), broadnosed pipefish (*Syngnatus typhle*: Knutsen et al. 2022), corkwing wrasse ( *Symphodus melops* : Blanco Gonzalez et al. 2016), European sprat ( *Sprattus sprattus* : Quintela et al. 2020), goldsinny wrasse ( *Ctenolabrus rupestris* : Jansson, André, et al. 2023), lumpfish ( *Cyclopterus lumpus* : Jansson, Faust, et al. 2023), common periwinkle ( *Littorina littorea* : Saha et al. 2025), northern shrimp ( *Pandalus borealis* : Knutsen et al. 2015), and sugar kelp (*Saccharina latissima*: Ribeiro et al. 2022). These species were chosen among commonly occurring organisms in the Norwegian coastal ecosystem to represent a wide range of trophic levels and life histories, and for their availability and ease of sampling, ensuring reasonable sample sizes over the entire study area, covering approximately 700 km of coastline. Most of the species were collected from the same or from closely situated locations, providing an opportunity to assess the drivers of gene flow with a consistent sampling design with regard to spatial range and ocean currents.

## 2 Material and Methods

**FIGURE 1:**
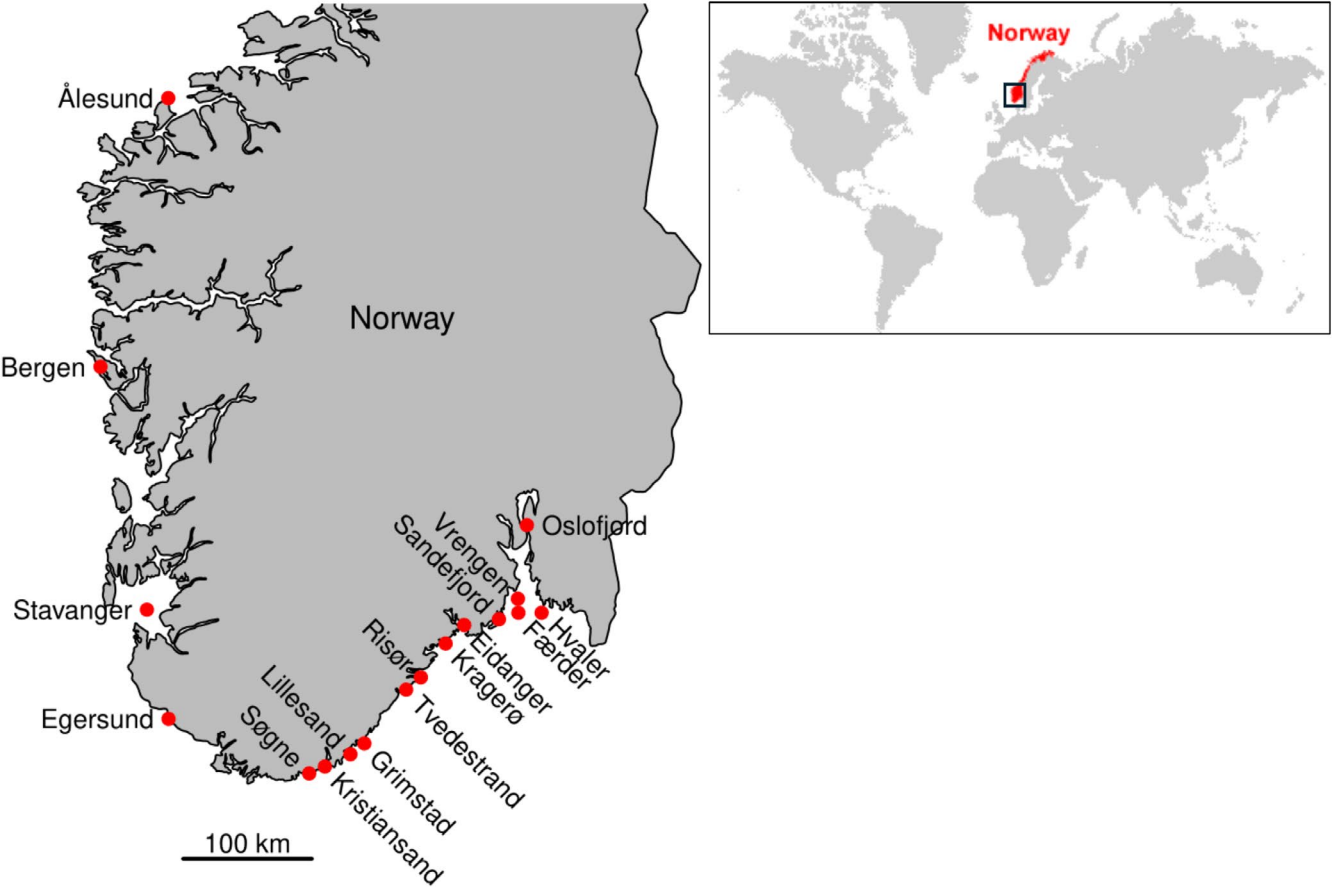
Study region in southern Norway showing the sampling locations for the corkwing wrasse, black goby, pipefish and common periwinkle. The other species were collected from closely situated sites from the same region (see Figure S1). Sample sites Ålesund, Bergen, Stavanger, and Egersund represent the west coast, the others the Skagerrak coast.

This investigation was conducted along the southern (Skagerrak) and western coasts of Norway (Figure 1; Figure S1). We reiterate some findings of the published studies and conduct additional analyses focusing on comparison among species. PLD estimates, collected from the literature, vary among species (summarized in Table 1) and allow for comparing genetic estimates of gene flow among species that differ in this key life‐history trait of supposed high relevance for the species' dispersal ability. For sugar kelp, pelagic propagule duration was used as this species is spore producing. Pipefish have parental (male) care and do not have free‐floating pelagic larvae, and so the PLD was set to 0 for this species, representing an extreme in the range of the PLD parameter. The other extreme is represented by the shrimp with a mean PLD of 68 days (range: 45–90). Methods of DNA extraction and genotyping are reported for each species in the publications cited in Table 1.

**TABLE 1:** Pelagic larval duration (PLD) in the studied 10 species.

Species	Min PLD	Mean PLD	Max PLD	Citation for PLD	Marker system (w/citation)	IBD slope	RMSE	Global *F*_ST_
Ballan wrasse	25	33	40	Sayer et al. 1996	SNP (Seljestad et al. 2020)	5.4E‐5	0.0073	0.0107
Black goby	14	21	28	e.g., Planes 1998; Knutsen et al. 2022	SNP (Catarino et al. 2022)	7.2E‐5	0.0115	0.0153
Broadnosed pipefish	0^a^	0^a^	0^a^	e.g., Knutsen et al. 2022	SNP (Knutsen et al. 2022)	1.0E‐4	0.0224	0.0337
Corkwing wrasse	14	21	28	Darwall et al. 1992; Knutsen et al. 2013	Microsatellites (Blanco Gonzalez et al. 2016)	1.6E‐4	0.0465	0.0506
European Sprat	30	45	60	Munk 1991	SNP (Quintela et al. 2020)	4.0E‐6	0.0033	0.0028
Goldsinny wrasse	30	38	45	Sayer et al. 1996	SNP (Jansson, André, et al. 2023)	< 1.0E‐6	0.0026	0.0036
Lumpfish	28	42	56	Davenport 1985	SNP (Jansson, Faust, et al. 2023)	< 1.0E‐6	0.0148	0.0139
Periwinkle	28	39	49	Johannesson 1988; Reid and Gofas 1996; Williams 1964	SNP (Saha et al. 2025)	1.0E‐5	0.0013	0.0011
Shrimp	45	68	90	e.g., Shumway 1985; Knutsen et al. 2015	Microsatellites (Knutsen et al. 2015)	1.0E‐6	0.0013	0.0009
Sugar kelp^b^	1	3	5	e.g., Mooney et al. 2018	Microsatellites (Ribeiro et al. 2022)	8.2E‐5	0.0346	0.0401

*Note:* Minimum, mean, and maximum reported PLD (days) are provided with their citations from literature. Estimated Isolation‐by‐distance (IBD) slope, and global *F*_ST_ values are provided in the seventh, and ninth column, respectively. The estimated root mean square errors of the regression line (RMSE) are presented in the eighth column.

^a^ Pipefish has no pelagic larval phase.

^b^ Sugar kelp's pelagic propagule duration is used as they produce spores.

For all 10 species, genetic differentiation were estimated in terms of *F*_ST_ (Weir and Cockerham 1984), using published pairwise *F*_ST_‐tables based on neutral genetic markers. The relationship between the linearized genetic distance, i.e., *F*_ST_/(1−*F*_ST_), and geographical distance was estimated for each species using linear regression in R (R Core Team 2024). Here, we used geographical distances over water, i.e., along the coast, among sample collections calculated using the R package MARMAP (Pante and Simon‐Bouhet 2013) with bathymetric data from the ETOPO1 database (Amante and Eakins 2009) hosted by NOAA (https://www.ngdc.noaa.gov/mgg/global/relief/ETOPO1/docs/ETOPO1pdf). To avoid samples on land due to limited resolution of the bathymetric data in coastal waters, we occasionally did slight adjustments of sampling positions (i.e., moving positions outward from the coast) before calculating distances. Distances along the coast follow closely the prevailing ocean currents in this region, flowing westward in the Skagerrak and northward along the western coast. Finally, we calculated linear regression of the 10 species' published PLD on the respective estimated IBD slope with the lm function in R. This regression analysis was repeated for minimum, maximum, and mean PLD‐estimates. The fit to a linear regression was calculated by the root mean square error of linear predictions relative to observed values: RMSE = sqrt (mean (observed—predicted)^2^).

## 3 Results

**FIGURE 2:**
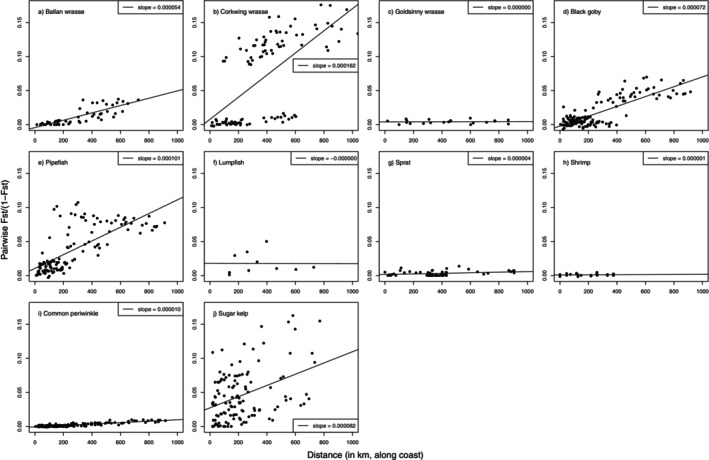
Results from IBD tests in 10 studied species (a–j). For each species dots represent sample pairs with geographic distance along the coast on the horizontal axis and genetic divergence, *F*_ST_/(1−*F*_ST_), along the vertical axis. Lines represent linear regressions with given slopes. See Table 1 for root mean square errors, RMSE, for the linear fits.

Low to moderate levels of genetic differentiation were observed in all 10 species in the studied region, with global *F*_ST_ over the study area ranging from 0.0009 to 0.056 (Table 1). Most species displayed an increase in pairwise *F*_ST_ with geographic distance between sample sites, i.e., an isolation‐by‐distance (IBD) trend (Figure 2; Table S1). However, the magnitude of this trend, as estimated by the linear regression slopes, varied greatly among species, from zero or nearly so (lumpfish, shrimp, and goldsinny wrasse) to a rather steep slope with *F*_ST_ increasing by 0.005 or more per 100 km (ballan wrasse, black goby, pipefish and corkwing wrasse).

**FIGURE 3:**
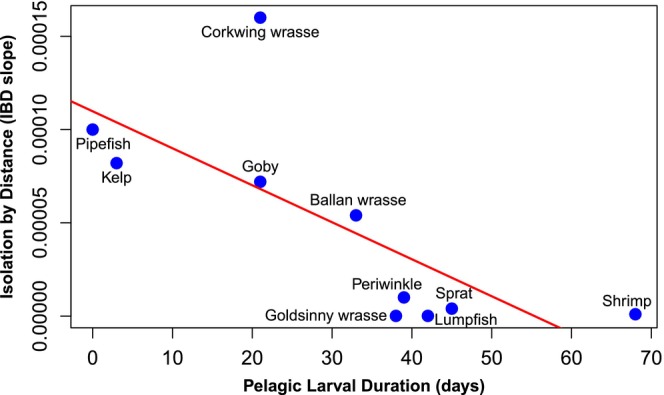
Relationship between IBD slope and mean pelagic larval duration (PLD) across 10 coastal species. A significant correlation was observed (*p* = 0.015, *r* ^2^ = 0.542). Excluding the corkwing wrasse increases the explained variation to *r* ^2^ = 0.79, indicating that PLD accounts for a substantial portion of the variation in IBD slope.

Linear regression supported a significant association between species' PLD and the estimated IBD slope (Figure 3, Table 1), which held no matter minimum PLD (*p* = 0.012, *r* ^2^ = 0.565), maximum PLD (*p* = 0.0195, *r* ^2^ = 0.515) or mean PLD (*p* = 0.015, *r* ^2^ = 0.542) was used. Most species can be described as having a reasonable linear trend in *F*_ST_ with distance, as judged by the visual plots (Figure 2) and the associated RMSE (Table 1). The corkwing wrasse, however, displayed a sharp genetic discontinuity and had the steepest IBD slope, standing out as an outlier in the regression. When excluded, the model fit improved (*r* ^2^ = 0.79). Pipefish, lacking a pelagic larval stage, had the second highest IBD slope (1.0 × 10^−4^), while the remaining eight species ranged from < 1.0 × 10^−6^ to 8.2 × 10^−5^.

## 4 Discussion

We found a significant correlation (*p* < 0.05) between species' PLD and estimated IBD slopes (Figure 3). This relationship remains consistent regardless of whether minimum, maximum, or mean PLD was used. This result verifies that PLD is an important dispersal mechanism in coastal species and that PLD in combination with the isolating effect of geographical distance jointly is a primary predictor of gene flow. Therefore, we reiterate earlier theoretical (Slatkin 1993; Rousset 1997) and empirical (Palumbi 2003; Selkoe and Toonen 2011) studies proposing that IBD slope is a useful metric for analyzing gene flow across species. The significant correlation between PLD and IBD slopes suggests that longer pelagic larval duration generally leads to greater dispersal and more gene flow (e.g., Bohonak 1999; Roberts 1997). We believe this verification of predictions can be ascribed to minimizing potential confounding factors by consistently sampling the same coastline across all 10 species, thus ensuring a robust dataset.

The present data show that PLD explains a large amount of variation of the IBD slope: *r* ^2^ = 0.54 in 10 species, rising to *r* ^2^ = 0.79 when excluding the corkwing wrasse. Our estimates are comparable to that of Selkoe and Toonen (2011) who conducted an investigation in a system of comparable size (~600 km). In line with Weersing and Toonen (2009), we find that minimum PLD estimates are better correlated with genetic metrics than mean estimates of PLD. However, unlike Weersing and Toonen (2009), who reported the correlation between PLD and global *F*_ST_, we report a correlation between PLD and the IBD slope. This is because global *F*_ST_ is sensitive to the size of the study area considered the “global” population and lacks a geographic context. In contrast, the stepping stone or isolation by distance model underlying the IBD relationship is more often a better representation of connectivity for marine species than the island model underlying global *F*_ST_ (Palumbi 2003; Selkoe and Toonen 2011).

Previous studies have identified two strongly differentiated populations of corkwing wrasse in the region, likely representing a secondary contact zone (Johannesson et al. 2020; Mattingsdal et al. 2020), which may explain why this species appears as an outlier in the relationship between IBD slope and PLD across species (Figure 3). Another important aspect is species‐specific migration behaviour. For example, lumpfish ( *Cyclopterus lumpus* ) exhibit complex spawning migrations. It has been shown that individuals caught offshore can be genetically distinct from nearby conspecifics, while spawning lumpfish sampled closer to breeding areas tend to be more genetically similar and show isolation‐by‐distance (IBD) patterns. Thus, depending on where and when lumpfish are sampled, two very different genetic patterns can emerge (Pampoulie et al. 2014; Whittaker et al. 2018; Jansson, Faust, et al. 2023). Hence, PLD does of course not describe all aspects of a species' population genetic structure, as historical processes and contemporary environmental conditions can also strongly influence genetic patterns (e.g., Stanley et al. 2018).

Our findings reiterate that genetic data can be a useful proxy for coastal species currently lacking information on population dispersal. Dispersal rate reflects the extent of gene flow among populations which has both evolutionary and ecological consequences (Bullock et al. 2006; Faurby and Barber 2012). Population dispersal helps in shaping ecosystem and species dynamics by enabling exploration of new resources and escape from unfavourable environments, thereby enhancing genetic diversity and ecosystem resilience (Peniston et al. 2024). While information on PLD has so far been scarce, and further research is needed for extracting PLD for many species, advances in high‐throughput technologies now provide accessible tools for studying genetic patterns in nearly any species. These advances offer new opportunities to investigate population dispersal across diverse systems.

In conclusion, we have demonstrated a strongly significant correlation between pelagic larval duration (PLD) and genetic population structure estimates of gene flow (IBD slope) across 10 coastal species spanning multiple trophic levels, reinforcing the well‐established link between longer PLD and weaker genetic structuring in marine populations. The clear and consistent linear relationship identified in our study underscores the predictive power of PLD in shaping connectivity patterns with unprecedented resolution and robustness.

## Funding

This study was supported by Universitetet i Agder; Nærings‐ og Fiskeridepartementet; European Research Council, HORIZON programme, project MARHAB (#101135307); Interreg Öresund‐Kattegat‐Skagerrak, project BlueBioClimate.

## Conflicts of Interest

The authors declare no conflicts of interest.

## Supporting information


**Data S1:** eva70187‐sup‐0001‐DataS1.xlsx.
**Figure S1:** Sampling locations of the studied 10 species. For details, also see Figure 1 and relevant published articles listed in Table 1.
**TABLE S1:** Ten species Fst & distance matrix.

## Data Availability

The primary data used in this study are provided in the Supporting Information. Additional biological data and other Supporting Information for periwinkle are accessible via the Dryad repository: https://doi.org/10.5061/dryad.qrfj6q5sz.
